# Current Advances in Proton FLASH Radiotherapy in Abdominal Cancers

**DOI:** 10.3390/cancers18050758

**Published:** 2026-02-27

**Authors:** Xiao Wang, Yin Zhang, Xinxin Zhang, Zhenyu Xiong, Keying Xu, Ning J. Yue, Chi Ma

**Affiliations:** Department of Radiation Oncology, Rutgers Cancer Institute, Rutgers Robert Wood Johnson Medical School, New Brunswick, NJ 08901, USA; xw240@cinj.rutgers.edu (X.W.); xz562@cinj.rutgers.edu (X.Z.); zx183@cinj.rutgers.edu (Z.X.); kx57@cinj.rutgers.edu (K.X.); yuenj@cinj.rutgers.edu (N.J.Y.); cm1276@cinj.rutgers.edu (C.M.)

**Keywords:** proton FLASH radiotherapy, proton therapy, abdominal cancer, gastrointestinal toxicity, ultra-high-dose-rate, Bragg peak, liver cancer

## Abstract

Proton FLASH radiotherapy represents a promising innovation in the treatment of abdominal cancers, offering the potential to expand the therapeutic window by delivering ultra-high dose rates (UHDR) that spare normal tissue while maintaining tumor control. This approach is particularly beneficial for gastrointestinal (GI) tumors, where radiation dose escalation is often limited by the radiosensitivity of nearby organs. This review explores recent preclinical, planning, and technical developments in proton FLASH for abdominal sites and outlines the challenges and future directions for clinical translation. We reviewed the available published literature on proton FLASH radiotherapy, with a focus on abdominal applications. Recent preclinical studies in abdominal models have shown encouraging, though inconsistent, evidence of reduced toxicity with proton FLASH. Parallel advances in treatment planning have also demonstrated technical feasibility in achieving UHDR across complex abdominal geometries. However, key challenges remain, including variability in biological responses, lack of standardized dose-rate definitions, delivery system constraints, and the absence of robust clinical data. Although only limited clinical trials are currently underway, proton FLASH may enable safer hypofractionation, reirradiation, and dose escalation strategies in the future. Its successful clinical translation will require coordinated advances in biology, physics, and technology, supported by rigorous preclinical validation and carefully designed clinical trials.

## Simple Summary

Proton FLASH radiotherapy represents one of the most promising frontiers in modern radiation oncology, with the potential to dramatically expand the therapeutic ratio for abdominal cancers. The GI tract has emerged as a critical focus due to its high radiosensitivity and proximity to many abdominal tumors. Preclinical findings on abdominal FLASH effects are encouraging yet inconsistent. The successful clinical translation remains complex, requiring resolution of biological uncertainties, standardization of dose rate metrics, robust treatment planning integration, and validation through well-designed clinical trials. With continued innovation in delivery hardware, planning systems, and mechanistic modeling, FLASH may ultimately enable safer dose escalation or reirradiation in abdominal sites where conventional approaches fall short.

## 1. Introduction

Abdominal cancers, including gastrointestinal malignancies of the colorectum, stomach, liver, and pancreas, represent a major global health burden. According to GLOBOCAN 2022 [1], gastrointestinal cancers account for over one-quarter of global cancer incidence and nearly one-third of cancer-related mortality [2]. Colorectal, liver, stomach, and pancreatic cancers remain among the leading causes of cancer-related death worldwide, with liver and pancreatic cancers projected to continue rising in incidence and mortality over the coming decades [3,4,5]. This substantial disease burden underscores the need for continued innovation in radiation therapy strategies that improve tumor control while minimizing gastrointestinal toxicity.

Radiation therapy (RT) plays an important role in managing abdominal cancer. Advances like intensity-modulated radiation therapy (IMRT) and stereotactic body radiation therapy (SBRT) have improved tumor targeting while minimizing GI toxicity [6,7]. In pancreatic cancer, dose escalation with ablative RT significantly prolongs local control and survival compared with conventional RT [8]. Selective internal radiation therapy (SIRT) for liver metastases from colorectal adenocarcinoma provides high local control with acceptable hepatic toxicity [9]. Proton therapy has been utilized in reirradiation in GI cancer, further reducing dose to adjacent bowel and stomach, enhancing safety in upper-abdominal tumors [10,11]. Continued innovations in adaptive RT and image guidance are reshaping the precision and efficacy of abdominal cancer therapy [12,13].

Conventional photon-based radiotherapy, despite advances in image guidance and intensity modulation, is limited by the proximity of critical structures that constrain dose escalation to tumor targets. Abdominal cancers are often situated near dose-limiting organs such as the small intestine, stomach, kidneys, and spinal cord, which possess low radiation tolerance and high radiosensitivity [14,15]. Even with highly conformal techniques such as volumetric modulated arc therapy (VMAT) or SBRT, the risk of radiation-induced gastritis (RIG), radiation enteritis, or radiation-induced liver disease (RILD) remains significant. It is particularly pronounced in hypofractionated regimens that deliver higher per-fraction doses [16,17,18]. Moreover, motion from respiration and peristalsis introduces uncertainties in target localization, further limiting achievable dose conformity [19,20,21].

Proton therapy has long been considered an attractive alternative for treating abdominal cancers due to its distinct Bragg peak in depth dose curve, enabling highly conformal delivery of radiation while sparing adjacent organs at risk (OARs) [22,23]. Proton beams can reach deep-seated abdominal targets, such as pancreatic and hepatic lesions, while confining dose to a finite depth, limiting collateral exposure to bowel and kidneys [24,25]. Clinical experience in hepatocellular carcinoma has demonstrated escalated dose to tumor, reduced mean doses to OARs and lower rates of hepatotoxicity compared with photon therapy [26,27]. However, for conventional dose rate proton therapy, normal-tissue toxicity remains a persistent barrier to further dose escalation, especially for liver and pancreas treatments [28,29,30].

To address this challenge, ultra-high-dose-rate (UHDR) proton therapy, commonly referred to as proton FLASH, has emerged as a promising alternative capable of producing additional biological sparing effects beyond geometric dose conformity [31,32,33]. By delivering radiation at UHDR, typically exceeding 40 Gy/s, FLASH has been shown in preclinical models to spare normal tissue while maintaining tumor control. This effect has been hypothesized to involve transient oxygen depletion and reduced reactive oxygen species formation [34,35]. These proposed mechanisms could be especially advantageous in the abdomen, where small bowel mucosa, liver parenchyma, and renal cortex are highly perfused and oxygen-dependent, yet prone to radiation-induced oxidative stress [36,37]. Indeed, studies using electron FLASH in abdominal irradiation have demonstrated significant GI tissue sparing without compromising tumor control in ovarian cancer mouse models [38,39].

This narrative review was based on a structured search of the PubMed database focusing on proton FLASH UHDR radiotherapy with relevance to abdominal organs. Search terms included combinations of “proton FLASH” or “UHDR” with anatomical keywords such as “abdominal,” “liver,” “pancreas,” “stomach,” “intestine,” “colorectal,” “kidney,” and “gallbladder.” Publications up until early 2025 were reviewed. Preclinical biological studies, treatment planning and dosimetric investigations, and early translational reports were included when relevant. For several abdominal organs, limited or no proton FLASH–specific studies were identified, highlighting existing gaps in the literature. This review was intended to be a focused narrative synthesis of available evidence, technical feasibility, limitations, and future research directions.

## 2. Current Development and Challenges

### 2.1. Preclinical Biology of Proton FLASH in the Abdomen

Over the past decade, animal models, primarily mice and zebrafish, have served as essential platforms for studying the FLASH effect. These models enable controlled investigation of tissue responses and radiobiological processes under UHDR conditions [40]. Multiple abdominal irradiation experiments involving proton beams have been reported, with interesting divergence in biological outcomes across studies.

Early preclinical work by Zhang et al. established a double-scattering proton system capable of reaching 120–138 Gy/s, allowing for reproducible small animal exposures at the entrance plateau region of the proton beam [35]. Using this system, mice receiving abdominal irradiation demonstrated better tolerance and lower mortality compared to conventional dose rates. These findings suggested tissue sparing at dose rates exceeding 100 Gy/s. Similarly, Diffenderfer et al. created a novel double-scattering system using single pencil beam, delivering uniform fields with dose rate of 60–100 Gy/s, and validated reduced acute and chronic GI toxicity and preserved crypt cell proliferation in murine models, confirming the biological feasibility of FLASH delivery in proton systems [41]. Evans et al. provided the first direct demonstration of the FLASH effect within the spread-out Bragg peak (SOBP) in abdominally irradiated mice [42]. Using 100 Gy/s synchrocyclotron beams, they observed increased LD50 values and 10–20% survival benefits compared with conventional dose rates, establishing that the advantage of Bragg peak can be combined with the normal tissue sparing benefits of FLASH treatments. Kim et al. investigated mouse abdominal irradiation at 15 Gy using both entrance plateau and SOBP regions [43]. They demonstrated that both the transmission beam technique using entrance plateau and the SOBP technique proton FLASH (107–108 Gy/s) preserved more regenerating intestinal crypts and showed similar tumor growth control compared to conventional dose rate proton (~0.8 Gy/s). The transmission beam technique and the SOBP technique showed no difference in tumor control, but using SOBP could further improve dose conformality and normal tissue sparing.

**Figure 1:**
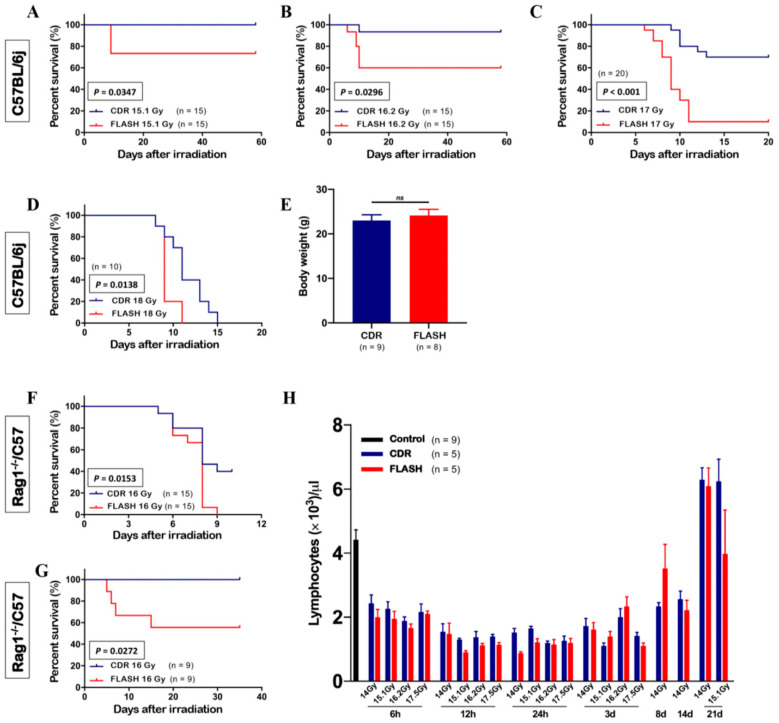
(**A**–**H**) Survival curves of C57BL/6j mice after FLASH and CDR (conventional dose rate) irradiation at different dose levels: (**A**) 15.1 Gy; (**B**) 16.2 Gy; (**C**) 17 Gy; (**D**) 18 Gy. (**E**) Body weight of C57BL/6j mice at 280 days post exposure. (**F**,**G**) Survival curves of Rag1^−/−^/C57 mice with different body weights ((**F**) 18.8 g; (**G**) 24.5 g) following 16 Gy irradiation. (**H**) Number of circulating lymphocytes in C57BL/6j mice at different timepoints. ns, not significant. Reprinted from [44], licensed under CC BY.

While several investigations reported normal tissue protection, others failed to replicate this effect. Zhang et al. found no intestinal sparing and even higher mortality compared to conventional dose rate irradiation in both C57BL/6j and immunodeficient Rag1^−/−^/C57 mice with partial abdominal irradiation at 120 Gy/s [44]. The beam was delivered to a 16 × 12 mm^2^ elliptical field covering approximately 60% of the abdomen, with mice placed in the entrance plateau region of the beam. Their resulting survival curves of C57BL/6j mice after FLASH and conventional dose rate irradiation at different dose levels are shown in Figure 1. Their findings suggest that the FLASH effect may not occur reliably in partial abdominal proton irradiation, and could depend on factors such as total irradiated volume, beam structure, tissue type, and experimental setup. The absence of protective effects in both immunocompetent and immunodeficient mice also challenges hypotheses that attribute the FLASH effect to lymphocyte sparing. Later, Bell et al. reported that whole-abdomen FLASH irradiation using pencil beam scanning (PBS) led to increased acute lethality and no histologic evidence of intestinal sparing [45]. C57BL/6J mice treated with 14 Gy at 80–100 Gy/s exhibited significantly lower survival compared to conventional proton therapy at 0.6 Gy/s, suggesting large field, high-dose, single-fraction PBS treatments may mitigate the FLASH advantage. Similarly, Liu et al. compared synchrotron-based proton FLASH (150 Gy/s for transmission beam, 230 Gy/s for SOBP) with conventional proton (0.2 Gy/s for transmission beam, 0.3 Gy/s for SOBP) and electron FLASH (188–205 Gy/s), and found proton FLASH irradiation resulted in greater GI toxicity and mortality compared to conventional proton therapy, with fewer regenerating crypts observed, unlike the protective response seen with electron FLASH [46]. Although no significant differences were observed in crypt regeneration between the transmission beam technique and the SOBP technique at the same dose for both proton FLASH and conventional proton, survival was lower with the SOBP technique under proton FLASH conditions. The findings suggest that FLASH effect is not solely dependent on mean dose rate but also varies by radiation modality, radiation type, and beam parameters.

Taken together, preclinical studies of proton FLASH in abdominal irradiation reveal a highly heterogeneous biological response. While several investigations demonstrate reduced gastrointestinal toxicity and preserved crypt regeneration under UHDR conditions, others report no sparing or even increased lethality. These discrepancies reveal that not all forms of UHDR proton delivery yield a biologically favorable outcome. Key experimental parameters and biological outcomes of representative preclinical proton FLASH studies involving abdominal irradiation are summarized in Table 1. Several factors may explain the divergent findings. The protective mechanisms observed in brain and lung may not extend to the highly perfused, rapidly proliferating intestine, where transient hypoxia may be insufficient to trigger the sparing effect associated with oxygen depletion [47,48]. Moreover, the different delivery pattern of proton beams, like continuous beam from cyclotron or pulsed beam from synchrotron, introduces variability in instantaneous dose rates, complicating the reproducibility of biological responses to FLASH irradiation [33,49]. The variability of gastrointestinal outcomes across proton FLASH studies highlights the possibility that these mechanisms may be context dependent, requiring specific dose-rate thresholds, irradiation volumes, or delivery patterns that are not consistently achieved in abdominal irradiation.

### 2.2. Planning Strategies and Challenges of Proton FLASH for Abdominal Sites

While the preclinical biology studies showed promising results, planning studies are essential in proton FLASH therapy to determine whether clinically deliverable beam configurations can achieve both conformal dose distributions and UHDR across complex anatomies such as the abdomen. The successful implementation of proton FLASH therapy requires rethinking conventional treatment planning approaches due to limitations in beam delivery speed, energy switching times, and spot scanning mechanics. Key planning considerations include simplifying field arrangements (e.g., transmission beams), maximizing per-field dose to ensure UHDR, and adopting standardized dose rate definitions to consistently evaluate FLASH eligibility across techniques and systems [50].

Treatment planning in abdominal cancers, particularly liver tumors, is gaining momentum, with numerous technical and clinical challenges. Several planning studies have proposed novel techniques to overcome dose conformity, delivery limitations, and optimization constraints in proton FLASH, particularly using pencil beam scanning PBS approaches.

**Figure 2:**
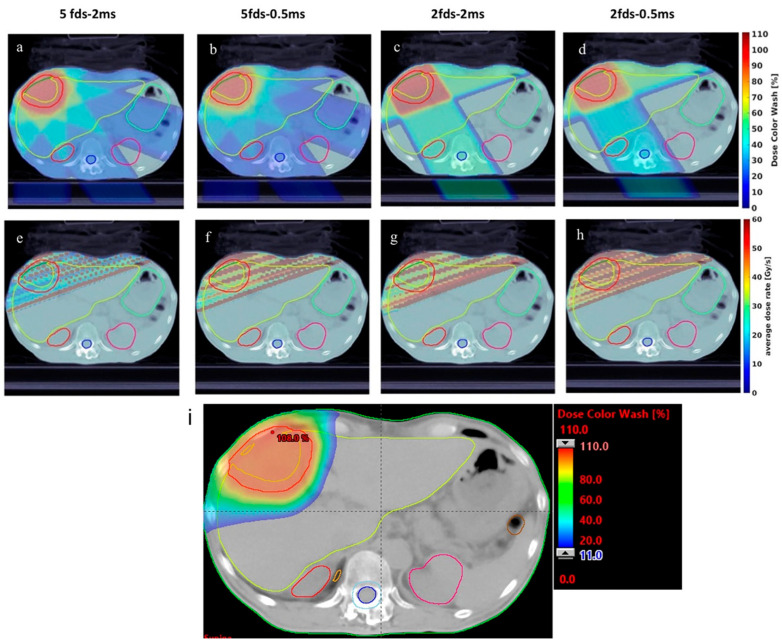
One example of the dose distribution for a single fraction: (**a**) 5 fields-2 ms minimum spot time, (**b**) 5 fields-0.5 ms, (**c**) 2 fields-2 ms, and (**d**) 2 fields-0.5 ms liver transmission plans, overlaid the CT images. The dose rate distribution for a single fraction: (**e**) 5 fields-2 ms minimum spot time, (**f**) 5 fields-0.5 ms, (**g**) 2 fields-2 ms, and (**h**) 2 fields-0.5 ms liver transmission plans, overlaid the CT images. (**i**) The 2D dose distribution of the SBRT plan for the same patient. Reprinted from [51], licensed under CC BY.

The earliest efforts in planning for abdominal proton FLASH therapy centered around transmission beam techniques due to their simplicity in achieving UHDR. Wei et al. conducted one of the first feasibility studies applying single-energy transmission PBS FLASH for hypofractionated liver cancer treatment using 240 MeV proton beams [51]. Their in-house treatment planning system (TPS) optimized two- and five-field arrangements and evaluated FLASH dose rate coverage (V40Gy/s) under varying minimum spot times and monitor units (MU). An example of dose distributions of proton FLASH plans with different field arrangements and different minimum spot times compared with dose distribution of SBRT plan is shown in Figure 2. The study found that using fewer fields and shorter minimum spot time improved both dose conformity and FLASH coverage in organs at risk (OARs), although transmission beams inherently compromised dose conformality due to the absence of distal fall-off and delivering exit dose to normal tissues.

To address the dose conformity limitations of transmission FLASH, the same group later developed a method using single-energy Pristine Bragg Peak (SEPBP) beams with range modulation through a universal range shifter (URS) and field-specific range compensators [52]. This approach enabled inverse modulated proton therapy (IMPT) FLASH planning with Bragg peak placement at the distal edge of the target. Their planning study on liver SBRT cases demonstrated comparable target coverage to conventional IMPT while eliminating exit dose and achieving sufficient FLASH dose rates. However, the plans showed sensitivity to minimum MU settings, as higher MU values (800 MU/spot) improved FLASH coverage but increased dose heterogeneity, highlighting the trade-off between dose rate and conformity.

With this foundation developed in the in-house TPS, Bookbinder et al. implemented the SEPBP FLASH technique into a commercial TPS (Eclipse), significantly advancing the clinical translatability of conformal FLASH planning [53]. The SEPBP method used sparse spotmaps and high-MU thresholds to maintain FLASH dose rates while preserving target conformity and limiting exposure to OARs. In liver SBRT cases, FLASH coverage exceeded 98% in critical structures at 5 Gy thresholds, and target conformity remained comparable to conventional IMPT plans. This represented an important milestone toward integrating FLASH capabilities into routine clinical workflows.

In a separate approach as single-energy SOBP (SESOBP), Ma et al. developed a computational framework to directly design physical pin-ridge filters (pin-RFs) from 3D dose distributions generated by single-energy transmission PBS plans, demonstrating that these streamlined filters could accurately replicate IMPT dose profiles and achieve >80% FLASH dose-rate coverage on lung cases [54]. Building upon this, they refined the hardware-assisted method for liver stereotactic ablative body radiotherapy (SABR) [55]. Their framework translated intermediate IMPT plans into physical pin-RF designs assembled from reusable components, allowing for delivery of FLASH-eligible single-energy PBS plans without compromising dose conformity. The study compared two-beam and three-beam configurations, finding that the simpler two-beam plans yielded better FLASH sparing at 5 Gy dose-rate thresholds, while three-beam setups degraded FLASH benefits due to lower per-beam doses. This indicated the importance of optimizing beam geometry not only for dose but also for FLASH effectiveness.

Another planning strategy explores arc-based proton FLASH therapy. Liu et al. developed SPLASH (SPArc + FLASH), a novel treatment planning framework that integrates FLASH dose rate optimization into spot-scanning proton arc therapy (SPArc) [56]. The key innovation of SPLASH lies in its voxel-level inverse optimization of both dose and dose rate using energy-modulated Bragg peak beams, rather than fixed-energy transmission beams, enabling voxel-level control without the need for patient-specific ridge filters. The technique modulates beam current and spot weights dynamically along the gantry arc, ensuring that dose rate thresholds (e.g., ≥40 Gy/s) are met within the clinical constraints of delivery systems. SPLASH demonstrated significantly improved FLASH dose-rate coverage and dose conformity in liver case compared to both IMPT and non-FLASH SPArc plans, without requiring additional hardware such as ridge filters. The study establishes SPLASH as a clinically deliverable and robust solution for FLASH treatment planning using full energy modulation and arc therapy.

Most recently, Rothwell et al. proposed Proton FLASH-Arc Therapy (PFAT) as a conceptual and simulation-based feasibility study aimed at achieving FLASH dose rates in the clinic using monoenergetic proton arcs [57]. Unlike prior approaches that focused on FLASH within the tumor, PFAT uses a simplified arc delivery scheme with fixed energy (transmission beams) and spatial-temporal spot sequencing to spatially fractionate dose delivery to normal tissues while concentrating linear energy transfer (LET) in the tumor. The method avoids full energy modulation, focusing instead on delivering ultrahigh dose rates to normal tissues by rotating the gantry and sequencing beamlets so that each healthy voxel receives only one brief, high-intensity dose during a single arc pass. While PFAT achieved acceptable target coverage and showed favorable FLASH dose-rate distributions in abdominal phantom, it does not currently support inverse planning or full clinical implementation. It serves more as a conceptual framework for biologically optimized FLASH delivery than a direct planning tool ready for deployment.

Collectively, proton FLASH planning studies demonstrate that achieving UHDR in abdominal targets is technically feasible, but often at the expense of traditional dosimetric advantages. Transmission beam approaches reliably achieve FLASH dose rates but compromise distal sparing, whereas Bragg-peak–based and hardware-assisted techniques improve conformity while tightening delivery constraints. Across studies, a consistent trend emerges in which fewer fields and higher per-field doses improve FLASH coverage but reduce plan robustness. These findings highlight an inherent trade-off between dose conformity, dose-rate eligibility, and clinical practicality that remains unresolved.

Despite these promising innovations, proton FLASH planning for abdominal treatments still faces unresolved challenges. One prominent issue is the lack of consensus on how dose rate should be defined and quantified. Multiple dose rate metrics, including average dose rate (ADR) [58], dose-averaged dose rate (DADR) [59], and dose-threshold dose rate (DTDR) [60], yield inconsistent estimates of FLASH coverage. These inconsistencies complicate both the optimization process and the clinical interpretation of treatment plans [52,61,62,63]. Additionally, delivery hardware limitations, such as energy layer switching times and beam current constraints, restrict the achievable dose rates for large targets or complex geometries [64]. It is important to note that many proton FLASH planning studies rely on in-house treatment planning systems or idealized delivery conditions that may not be directly achievable on current clinical proton platforms. Assumptions regarding beam current, spot dwell time, energy layer switching speed, and dose-rate control often exceed the capabilities of commercially available systems. As a result, reported FLASH coverage and dose-rate metrics may represent best-case scenarios rather than immediately applicable clinical solutions. It’s worth noting that, while these studies demonstrate that FLASH-eligible dose-rate distributions can be achieved in abdominal geometries using a variety of planning strategies, technical feasibility alone does not imply biological sparing or clinical benefit. The presence of UHDR within a treatment plan should therefore be interpreted as an enabling condition rather than validation of a FLASH effect in GI tissues.

## 3. Future Directions of Improvement

While encouraging results have emerged from preclinical models and early planning studies, the field remains in a developmental phase. Future progress in proton FLASH radiotherapy for abdominal applications depends on coordinated advances across several domains. Based on current evidence, priorities can be broadly ordered as biological validation, dose-rate metric standardization, treatment planning system development, delivery hardware and quality assurance, and finally clinical trials.

### 3.1. Biological Validation and Understanding of the FLASH Effect in Abdominal Organs

A significant area of future work is refining our biological understanding of the FLASH effect in abdominal organs, particularly the gastrointestinal (GI) tract. While multiple studies report tissue-sparing effects in skin [65,66,67], lung [68,69,70], and brain [70,71,72], results in the abdomen are mixed, as discussed in Section 2.1. While several studies demonstrate intestinal and hepatic sparing [35,41,42,43], others report neutral or even adverse effects [44,45,46]. The contradicting findings in abdominal tissue in the preclinical studies raise caution about extrapolating FLASH benefits to GI tissues [69,73], emphasizing the need for proposed mechanism studies including oxygen depletion, reactive oxygen species (ROS) signaling, and immune modulation in abdominal organs and tissue-specific thresholds for FLASH sparing [48,74]. Despite rapid progress in planning and delivery techniques, biological validation remains the limiting factor for abdominal FLASH translation. In contrast to lung and brain models, GI tissues have demonstrated highly variable and sometimes adverse responses to proton FLASH irradiation. These findings suggest that achieving FLASH dose rates in treatment plans is a necessary but insufficient condition for toxicity reduction, reinforcing the need for tissue-specific biological thresholds and mechanistic validation.

The FLASH effect is not only governed by physical dose rate but also involves a complex interplay of biological effects including stem cell dynamics and immune repair signaling [75]. Yet many biological assumptions remain speculative [32,74]. Several mechanisms have been proposed to explain the FLASH effect, including transient oxygen depletion, reduced reactive oxygen species production, vascular preservation, and immune modulation. However, these mechanisms should be regarded as hypotheses rather than established explanations. Most available evidence has been derived from non-abdominal models, and conflicting preclinical GI results indicate that these mechanisms may not operate uniformly across tissues or radiation modalities. In particular, the absence of consistent gastrointestinal sparing in proton FLASH studies suggests that oxygen depletion or immune-mediated protection alone may be insufficient to explain observed outcomes. Lack of robust biological models impedes predictive modeling for treatment planning. Critical FLASH-modulating variables include instantaneous dose rate, total irradiation time (<200 ms), fractionation, and LET-dependent effects [61]. Understanding how these interact in abdominal organs, especially hypoxic tissues like the liver or gut, is critical to clarify where and when FLASH may offer benefits [76].

Upcoming studies should incorporate both acute and late toxicity measures, including intestinal crypt survival, vascular permeability, inflammatory cytokine profiling, and long-term fibrosis [69,77]. The integration of in vivo oxygen probes and microdosimetric LET mapping will further elucidate correlations between temporal dose structure and biological response [78,79]. Additionally, the dose-modifying factor (DMF), which quantifies the biological effect difference between FLASH and conventional doses, is not yet standardized, making inter-study comparisons difficult [70,76]. Future studies must establish biological endpoints specific to abdominal tissues and clarify dose thresholds, particularly given that the current FLASH knowledge is heavily influenced by preclinical data from non-abdominal sites [80].

### 3.2. Standardization of FLASH Dose Rate Metrics

One of the most significant barriers to reproducibility, biological interpretation, and clinical translation of FLASH radiotherapy is the absence of standardized dose rate definitions. Various definitions, such as ADR, DADR, and DTDR, are used inconsistently across preclinical and planning studies, each reflecting different aspects of temporal and spatial dose delivery. ADR is typically computed by dividing total delivered dose by the overall irradiation time, which can underestimate the dose rate, particularly in regions receiving low cumulative dose or located at the periphery of the beam. In contrast, DADR weights dose rate by voxel-level dose contribution, while DTDR considers only dose delivery above a predefined threshold (e.g., 40 Gy/s). Kang et al. demonstrated that ADR may significantly undervalue the effective dose rate compared to DADR or DTDR, potentially leading to underestimation of FLASH coverage in organs at risk (OARs), especially at the edges of treatment fields [60]. This discrepancy can have direct implications on clinical decision-making, particularly when dose rate is used as a surrogate for biological sparing.

The situation is further complicated in PBS, where instantaneous dose rate at the spot level may be orders of magnitude higher than voxel-averaged or delivery-averaged dose rate due to spot sequencing, inter-spot pauses, and energy layer switching. Consequently, reported dose rates must be interpreted in the context of how temporal structure is incorporated into the calculation, as instantaneous and averaged dose-rate metrics are not interchangeable and may have different biological implications. Deffet et al. highlighted that dose rate estimates can vary substantially depending on whether spot timing, inter-spot pauses, or machine-specific parameters are incorporated into the metric [63]. The use of inconsistent dose-rate definitions across FLASH studies fundamentally limits cross-study comparison and reproducibility. Identical treatment plans may be classified as FLASH-eligible or non-FLASH depending on whether ADR, DADR, or DTDR are applied. This variability complicates interpretation of biological outcomes, obscures dose–response relationships and hinders the development of unified planning objectives. Without consensus on standardized, biologically meaningful dose-rate metrics, it remains difficult to translate preclinical findings into clinical protocols or to design robust multicenter trials. Some conflicting reports of preclinical GI FLASH effects may partially reflect differences in how dose rate was defined and quantified rather than true biological disagreement.

Future efforts should focus on defining clinically meaningful and practically measurable dose rate metrics that correlate with biological outcomes and are compatible with current delivery systems. In parallel, commercial treatment planning systems must be updated to support voxel-level mapping of standardized dose rate parameters, thereby enabling robust integration of FLASH optimization into routine clinical workflows.

### 3.3. TPS Development and Directions of Capabilities

Since most of the planning studies were conducted with in-house platforms, the development of commercial TPS is in great need of proton FLASH implementation. The reliance on in-house TPS platforms underscores the current lack of FLASH-ready capabilities in commercial systems, including voxel-level dose-rate calculation, inverse FLASH optimization, and delivery constraints. Without vendor-supported solutions, routine clinical planning, verification, and multicenter standardization remain challenging. Future development of TPS should include voxel-wise dose rate mapping, inverse FLASH optimization, and adaptive re-planning to accommodate anatomical or beam delivery variation [81]. In addition, efforts to incorporate FLASH dose rate metrics into planning systems are critical for standardizing the FLASH effect’s quantification.

While physical parameters dominate current FLASH planning, going forward, integrating biological factors, such as oxygen depletion, DNA repair kinetics, and LET dependence, into optimization may significantly improve patient-specific FLASH effectiveness [82]. Mechanistic models like the “FLASH Effectiveness Model” and “Sliding Window” dose-rate models offer a pathway to this integration, though they remain under validation [81,83]. Moreover, optimization frameworks must account for both biological parameters and machine delivery constraints to ensure feasibility.

Longer term, FLASH planning could be revolutionized by AI-assisted planning that integrates radiobiological parameters with historical patient data to recommend FLASH-eligible beam angles, spot maps, and dose regimes.

### 3.4. Hardware and Delivery Innovations

Technical constraints also limit the realization of consistent FLASH dose rates. Transmission proton beams easily achieve >40 Gy/s but sacrifice the dosimetric advantage of the Bragg peak, resulting in elevated exit doses to distal normal tissue [84]. Conversely, Bragg peak or SOBP delivery provides excellent conformity but struggles to maintain FLASH dose rates because of energy degradation, layer switching times, and beam current loss at lower energies [85]. Even with range shifters or compensators, achieving a consistent >40 Gy/s mean rate over an abdominal target remains challenging [52]. Moving forward, future directions include hardware acceleration for energy layer switching, increased beam current capabilities in clinical systems, and integration of ultra-fast raster scanning protocols [86].

In addition, successful clinical implementation will depend on rigorous verification and quality assurance (QA). Dedicated dosimetry tools capable of submillisecond temporal resolution and minimal recombination are being developed to validate instantaneous dose rate and total delivered dose [87,88]. FLASH-specific QA protocols should be integrated into clinical workflows to ensure safety and reproducibility. Additionally, FLASH planning approaches that require custom hardware or nonstandard delivery sequences introduce added complexity in treatment commissioning, quality assurance, and regulatory approval, further limiting near-term clinical implementation.

### 3.5. Clinical Trials and Translation to Clinical Practice

To ensure effective clinical translation, a concerted push for well-designed trials is essential. Currently, most proton FLASH studies are limited to feasibility and preclinical phases, with only two ongoing clinical trials on bone metastases [89,90]. Future clinical trials should be disease site-specific and require standardized dose rate definition and comparable planning strategies, as well as standardized biomarkers and protocols for toxicity and tumor control endpoints [91]. Such trials should not only validate clinical feasibility but also inform optimal fractionation and field design [92]. This may not happen in the near future, since clinical trials should build upon established animal models with known FLASH parameters, as discrepancies in GI toxicity across species have already emerged [40].

The successful clinical translation of proton FLASH for abdominal sites will likely begin with focal liver and pancreatic targets under controlled hypofractionated regimens. In addition, the combination of geometric precision and biological protection may allow higher fractional doses or re-irradiation in patients with recurrent or previously treated disease [11,93].

Integrating FLASH protocols into broader treatment paradigms, such as adaptive therapy and functional imaging, may expand clinical utility [94]. For instance, combining FLASH with real-time tumor motion tracking could be especially impactful for mobile abdominal organs. Additionally, future FLASH protocols may benefit from synergy with immunomodulation strategies, as preclinical evidence hints at unique immune-stimulatory properties of UHDR that could potentiate systemic response [92].

Importantly, uncertainties in biological response, lack of standardized dose-rate metrics, and planning and delivery system limitations are tightly coupled challenges, and progress in any single domain is unlikely to enable clinical translation in isolation. Among the future directions discussed, biological validation in GI tissues and standardization of dose-rate metrics appear to be the most probable and impactful near-term priorities. Inconsistent preclinical outcomes in abdominal organs suggest that without a clearer understanding of tissue-specific FLASH thresholds and biologically relevant dose-rate definitions, further advances in planning algorithms or delivery hardware alone are unlikely to yield reliable clinical benefit. Establishing reproducible biological endpoints and standardized dose-rate metrics would enable meaningful comparison across studies and provide the foundation upon which TPS development, hardware optimization, and eventual clinical trials can be built.

## 4. Conclusions

Proton FLASH radiotherapy represents one of the most promising frontiers in modern radiation oncology, with the potential to dramatically expand the therapeutic ratio for abdominal cancers. The GI tract has emerged as a critical focus due to its high radiosensitivity and proximity to many abdominal tumors. Preclinical findings on abdominal FLASH effects are encouraging yet inconsistent. Advances in treatment planning are expanding the feasibility of delivering UHDR without compromising conformity, yet significant barriers remain. To date, evidence supporting proton FLASH radiotherapy for abdominal cancers is confined to preclinical animal studies, and its safety and efficacy in humans remain unproven. Despite promising preclinical and technical developments, proton FLASH radiotherapy for abdominal cancers remains far from clinical applicability due to inconsistent biological evidence and the absence of standardized clinical protocols. With continued innovation in delivery hardware, planning systems, and mechanistic modeling, FLASH may ultimately enable safer dose escalation or reirradiation in abdominal sites where conventional approaches fall short.

## Figures and Tables

**Table 1:** Summarization of representative preclinical proton FLASH studies involving abdominal irradiation.

Study	Irradiated Volume	Beam Structure	Fractionation	Primary Biological Endpoints	FLASH Effect Observed?
Zhang et al. (2020) [35]	Partial abdomen	Double scattering, entrance plateau	Single fraction	Survival, body weight, late tissue response	Yes (improved survival, less weight loss, better intestinal repair)
Diffenderfer et al. (2020) [41]	Whole abdomen	Double scattering	Single fraction	Intestinal crypt survival, acute and late GI toxicity	Yes (reduced toxicity, preserved crypts)
Kim et al. (2021) [43]	Whole abdomen	Double scattering, entrance plateau vs. SOBP	Single fraction	Intestinal crypt regeneration, tumor growth control	Yes (crypt sparing with comparable tumor control)
Evans et al. (2022) [42]	Whole abdomen	Synchrocyclotron, SOBP	Single fraction	LD50, survival, body weight	Yes (increased LD50, improved survival, less weight loss)
Zhang et al. (2023) [44]	Partial abdomen (~60% abdominal volume)	Double scattering, entrance plateau	Single fraction	Survival, circulating lymphocyte counts, proliferating crypt cells, long-term intestinal muscularis externa thickness	No (no GI sparing, similar or worse survival)
Bell et al. (2025) [45]	Whole abdomen	Pencil beam scanning (PBS)	Single fraction	Acute lethality, intestinal histology	No (increased lethality, no GI sparing)
Liu et al. (2025) [46]	Whole abdomen	Synchrotron-based transmission vs. SOBP, Electron	Single fraction	Crypt survival, mortality	Proton: No (greater GI toxicity, worse survival); Electron: Yes

## Data Availability

All the data has been shared in this publication.
